# Performance of BIOFIRE FILMARRAY pneumonia panel in suspected pneumonia: insights from a real-world study

**DOI:** 10.1128/spectrum.00571-25

**Published:** 2025-05-22

**Authors:** Mona Mustafa Hellou, Abinash Virk, Angel P. Strasburg, William S. Harmsen, Paschalis Vergidis, Kirstin Kooda, Kami D. Kies, Alexander D. Donadio, Jay Mandrekar, Audrey N. Schuetz, Robin Patel

**Affiliations:** 1 Division of Clinical Microbiology, Department of Laboratory Medicine and Pathology, Mayo Clinic 4352, Rochester, Minnesota, USA; 2 Division of Public Health, Infectious Diseases, and Occupational Medicine, Department of Medicine, Mayo Clinic 6915, Rochester, Minnesota, USA; 3 Department of Biomedical Statistics and Informatics, Mayo Clinic 6915, Rochester, Minnesota, USA; 4 Department of Pharmacy, Mayo Clinic 6915 , Rochester, Minnesota, USA; MultiCare Health System, Tacoma, Washington, USA

**Keywords:** pneumonia, BIOFIRE, analytical performance, positive percent agreement, negative percent agreement, concordance

## Abstract

Pneumonia may be caused by a diverse group of microorganisms; however, a microbiologic diagnosis is not universally made. Molecular tests such as the BIOFIRE FILMARRAY Pneumonia Panel (BF-PP) offer the possibility of rapid identification of potential pneumonia pathogens. This retrospective analysis was conducted as a sub-study of a recently published randomized controlled trial. Specimens from patients with suspected pneumonia were tested by the BF-PP, with results compared to cultures. The overall BF-PP positivity rate was 44.8% (252/563), and was higher [64.6% (164/254)] in specimens from patients ultimately diagnosed with pneumonia. Discrepancies between the BF-PP and cultures were most common for *Streptococcus pneumoniae*, *Haemophilus influenzae*, *Moraxella catarrhalis,* and *Streptococcus agalactiae*. Positive percent agreement (PPA) and negative percent agreement (NPA) between the BF-PP and culture were 91.4% (CI 86.3%–94.9%) and 83.6% (CI 79.5%–87.2%), respectively. For most bacteria, a correlation between high genomic abundance (genomic copies/mL) reported from the BF-PP and culture growth abundance was found. In specimens from patients ultimately diagnosed with pneumonia, clinical consistency—assessed based on whether BF-PP results aligned with associated culture results—was 79.9% and was highest for bronchoalveolar lavage fluid specimens. The BF-PP has reliable analytical performance and offers the potential advantage over conventional cultures of providing higher detection rates and more rapid results. Interestingly, despite the use of this advanced molecular diagnostic tool, one-third of pneumonia cases remained without a microbiologic etiology.

**IMPORTANCE:**

This study evaluates the BIOFIRE FILMARRAY Pneumonia Panel by comparing its performance to conventional cultures in a real-world patient population (including patients ultimately diagnosed as not having pneumonia) using different types of specimens. Findings show a higher detection rate of microorganisms with the panel compared to cultures, suggesting that this test could aid in tailoring treatments for pneumonia. However, challenges remain and require further study, including distinguishing true pathogens from colonizing microorganisms and determinig why one-third of patients diagnosed with pneumonia still lacked a microbiologic etiology.

## INTRODUCTION

Pneumonia is a common cause of morbidity and mortality worldwide. Its heterogeneous etiology is changing (1) due to introduction of pneumococcal, influenza, respiratory syncytial virus (RSV) and other vaccines, and emergence of antibiotic-resistant bacteria (2). Antibiotic treatment remains largely empirical. Therefore, it is possible that patients with pneumonia receive antibiotics they do not need or do not receive antibiotics that may benefit them; inappropriate use of antibiotics drives increased resistance and microbiome disturbances (3). Rapid detection of causative pathogens in patients with pneumonia can potentially inform timely, targeted antibiotic treatment.

Conventional cultures are the most widely used method for microbiological diagnosis of pneumonia. However, they are labor-intensive, time-consuming, and have low (20%–40%) pathogen detection rates (4, 5). Furthermore, the effect of prior antibiotic use on cultures, along with contamination by normal microbiota and/or colonizing microorganisms, poses a challenge in accurately diagnosing true pathogens (6). These limitations underscore the need for diagnostic approaches with faster and more reliable performance.

The BIOFIRE FILMARRAY Pneumonia Panel (BF-PP) is a multiplex nucleic acid test that detects multiple bacteria and viruses, along with select genetic resistance determinants in respiratory specimens. It is an automated sample-to-answer system, with minimal hands-on time, providing results in about 75 minutes. The overall positivity rate for detecting at least one microorganism has been reported to reach 72% and 49% for sputum and bronchoalveolar lavage (BAL) fluid, respectively, based on analytical and clinical studies evaluating the panel’s performance for regulatory clearance (7, 8). However, whether this reflects higher detection of pathogens or microorganisms colonizing the respiratory tract is challenging to define.

In this study, the analytical performance of the BF-PP was compared to that of cultures, as a sub-study of a recently-published randomized controlled trial (RCT) evaluating the clinical utility of the assay in patients with suspected pneumonia (9).

## MATERIALS AND METHODS

This was a retrospective sub-study of a single center, open label, RCT (9), evaluating the clinical utility of the BF-PP compared to standard microbiologic testing on antibiotic use in a real-world hospital setting. The study was conducted at Mayo Clinic, Rochester, MN, from 2020 to 2022, in patients ≥ 18 years old hospitalized with suspected pneumonia. The Mayo Clinic Institutional Review Board approved the study protocol and a waiver of informed consent in accordance with 45 CFR 46.116, and a waiver of Health Insurance Portability and Accountability Act authorization in accordance with applicable regulations.

Respiratory specimens from eligible patients were randomly assigned to undergo testing with the BF-PP plus conventional culture and antimicrobial susceptibility testing (AST) (intervention arm) or conventional culture and AST alone (control arm). The current study assessed respiratory specimens from the intervention arm, including specimens from patients ultimately diagnosed with pneumonia and those subsequently considered not to have pneumonia. Results of the BF-PP were compared to those of cultures. Respiratory specimens included expectorated and induced sputum, tracheal secretions, and BAL fluid. Only sputum specimens passing quality standards for culture (i.e., using Gram stain) were accepted for testing with the BF-PP and culture (10). Specimens were cultured on blood, chocolate, and eosin methylene blue agar (Becton Dickinson) and incubated at 35°C for 2 days, with the first examination after 16–24 h of incubation. For cultures with growth, results were reported semi-quantitatively as +1, +2, +3, and +4.

The BF-PP detects 18 bacteria, 8 viruses, and 7 genetic resistance determinants. For *Acinetobacter calcoaceticus-baumannii* complex, *Enterobacter cloacae* complex, *Escherichia coli, Klebsiella aerogenes, Klebsiella oxytoca, Klebsiella pneumoniae* group, *Proteus* spp., *Pseudomonas aeruginosa, Serratia marcescens, Haemophilus influenzae, Moraxella catarrhalis, Streptococcus agalactiae, Streptococcus pneumoniae, Streptococcus pyogenes, and Staphylococcus aureus,* results are reported semi-quantitatively as 10^4^, 10^5^, 10^6^, and ≥10^7^ genomic copies/mL. *Chlamydia pneumoniae, Legionella pneumophila*, *Mycoplasma pneumoniae*, viruses (adenovirus, coronavirus, human metapneumovirus, human rhinovirus/enterovirus, influenza A virus, influenza B virus, parainfluenza virus, and respiratory syncytial virus), and resistance determinants (*bla*_CTX-M_, *bla*_IMP_, *bla*_KPC_, *bla*_NDM_, *bla*_OXA-48-like_, *bla*_VIM_, and *mecA/C* and MREJ) are reported qualitatively as positive or negative. The BF-PP was performed per package insert instructions, with no specimen processing prior to testing.

### Data and statistical analysis

The BF-PP positivity rate was calculated based on any bacterial or viral detection. Culture positivity rate was determined by the detection of bacterial growth. Cultures reported as usual respiratory microbiota or showing only fungal growth were not considered positive.

Concordance between the BF-PP and cultures was calculated for each bacterium by determining the number of specimens in which the bacterium was detected by the BF-PP and culture, divided by the total number of specimens in which that particular bacterium was detected by the BF-PP. Cultures reported by the microbiology laboratory as usual respiratory microbiota in which the BF-PP detected *Streptococcus pneumoniae*, *Haemophilus influenzae*, *Moraxella catarrhalis,* or *Streptococcus agalactiae* were re-evaluated to search for the detected species.

Performance metrics of positive percent agreement (PPA) and negative percent agreement (NPA) were used according to the FDA guidance on reporting diagnostic assay study results (11). PPA and NPA were calculated as follows: PPA, the number of concordant positive results divided by the total number of positive culture results. NPA, the number of concordant negative results divided by the total number of negative culture results. Estimates of the BF-PP PPA and NPA were calculated considering culture as the reference standard, thus only referring to bacteria. An exact binomial 95% confidence interval was also reported for each estimate. PPA, NPA, and positivity rate were compared using either chi-square or Fisher’s exact tests, as appropriate. A significance level of *α* = 0.05 was used. All statistical analyses were conducted using SAS version 9.4.

Since certain microorganisms were infrequently detected, results were aggregated as follows. The “usual respiratory microbiota group” (URM group) included *S. pneumoniae*, *H. influenzae*, *M. catarrhalis,* and *S. agalactiae*. The “non-fermenting Gram-negative bacilli group” included *P. aeruginosa* and *A. calcoaceticus-baumannii* complex. “Enterobacterales group” included *E. coli*, *E. cloacae* complex, *K. aerogenes*, *K. oxytoca*, *K. pneumoniae* group (hereafter referred to as *K. pneumoniae*), *Proteus* species, and *S. marcescens. S. aureus* was reported separately. PPA and NPA, stratified by clinical syndrome [community-acquired pneumonia (CAP), hospital-acquired pneumonia (HAP), ventilator-associated pneumonia (VAP)], and specimen type (sputum, induced sputum, tracheal secretions, and BAL fluid) within the pneumonia cohort were calculated for aggregated microorganisms.

Correlation between semiquantitative results (genomic abundance, categorized as 10^4^, 10^5^, 10^6^, and ≥10^7^ genomic copies/mL) and semiquantitative growth in cultures for bacterial groups was assessed as follows: genomic abundances of 10^4^ or 10^5^ were classified as low (<10^6^), while 10^6^ or ≥10^7^ were classified as high (≥10^6^). For culture growth, +1 and +2 were considered low growth, and +3 and +4 high growth.

Clinical consistency between the BF-PP and culture was calculated for specimens from the pneumonia cohort based on whether BF-PP aligned with culture results (including those for bacteria not detected by the panel) in terms of potential impact on clinical decision making, particularly antibiotic modification (Table S1).

Pneumonia was defined based on clinical and radiological findings and classified as CAP if contracted in the community, HAP if symptoms developed at least 48 hours after hospital admission, or VAP if onset occurred at least 48 hours after intubation.

Infection Other than Pneumonia or No Infection (OP-NI) was defined by the antimicrobial stewardship team or by the infectious diseases consultant overseeing patient treatment. Among subjects without pneumonia, diagnoses included non-pulmonary infection (e.g., intraabdominal infection), aspiration, empyema, lung abscess, fungal pneumonia, COVID-19 pneumonia (SARS-CoV-2 is not included in the BF-PP), viral upper respiratory tract infection, acute exacerbation of COPD, atelectasis, mucus plug, and pulmonary edema.

## RESULTS

In all, 563 specimens were included—162 sputum, 26 induced sputum, 198 tracheal secretions, and 177 BAL fluid specimens. Two hundred fifty-four specimens were from patients ultimately classified as having pneumonia and 309 from those ultimately classified as having OP-NI. Among the pneumonia specimens, 41.7% were associated with CAP, 38.6% with HAP, and 19.7% with VAP.

Overall positivity of the panel for the detection of at least one microorganism was 44.8% (252/563), being highest for induced sputum (69.2%) and lowest for BAL fluid (18.1%). The positivity rate for pneumonia-associated specimens was higher than for OP-NI specimens (64.6% vs 28.5%, respectively [*P* < 0.0001]), across most specimen types ([sputum, 62.9% vs 40.7%, *P* = 0.002], [induced sputum, 78.6% vs 58.3%, *P* = 0.4], [tracheal secretions, 73.1% vs 43.3%, *P* < 0.0001], [BAL fluid, 45.1% vs 7.1%, *P* < 0.0001], respectively) (Table 1). More than one microorganism was detected in 39.3% (99/252) of positive specimens. The maximum number of microorganisms identified in a single specimen was 6 (observed in 1 specimen). The most frequent microorganisms detected across all specimens were *S. aureus* (27.3%), *H. influenzae* (17.4%), *P. aeruginosa* (11.0%), and *S. agalactiae* (8.7%). In pneumonia-associated specimens, the most identified bacteria were *S. aureus* (26.5%), *H. influenzae* (14.3%), *P. aeruginosa* (10.9%), and *S. pneumoniae* (7.6%) (Table 2).

**TABLE 1:** BIOFIRE FILMARRAY pneumonia panel positivity rate for bacteria and/or viruses

	All samples	Sputum	Induced sputum	Tracheal secretions	Bronchoalveolar lavage fluid
Overall	252/563 (44.8%)	84/162 (51.9%)	18/26 (69.2%)	118/198 (59.6%)	32/177 (18.1%)
Pneumonia	164/254 (64.6%)	51/81 (62.9%)	11/14 (78.6%)	79/108 (73.1%)	23/51 (45.1%)
Community-acquired pneumonia	65/106 (61.3%)	32/53 (60.4%)	4/5 (80.0%)	20/29 (68.9%)	9/19 (47.4%)
Hospital-acquired pneumonia	62/98 (63.3%)	19/28 (67.9%)	7/9 (77.8%)	24/33 (72.7%)	12/28 (42.9%)
Ventilator-associated pneumonia	37/50 (74.0%)	Not applicable	Not applicable	35/46 (76.1%)	2/4 (50.0%)
Infection other than pneumonia or no infection	88/309 (28.5%)	33/81 (40.7%)	7/12 (58.3%)	39/90 (43.3%)	9/126 (7.1%)

**TABLE 2:** Distribution of bacteria detected by the BIOFIRE FILMARRAY pneumonia panel in respiratory specimens

Microorganism (*n*)	All specimens, *N* = 344	Pneumonia specimens, *N* = 238
*Staphylococcus aureus*	94 (27.3%)	63 (26.5%)
*Streptococcus pneumoniae*	20 (5.8%)	18 (7.6%)
*Haemophilus influenzae*	60 (17.4%)	34 (14.3%)
*Streptococcus agalactiae*	30 (8.7%)	16 (6.7%)
*Moraxella catarrhalis*	12 (3.5%)	11 (4.6%)
*Pseudomonas aeruginosa*	38 (11.0%)	26 (10.9%)
*Acinetobacter baumannii*	6 (1.7%)	5 (2.1%)
*Escherichia coli*	21 (6.1%)	17 (7.1%)
*Enterobacter cloacae*	13 (3.8%)	12 (5.0%)
*Klebsiella aerogenes*	6 (1.7%)	4 (1.7%)
*Klebsiella oxytoca*	6 (1.7%)	5 (2.1%)
*Klebsiella pneumoniae*	20 (5.8%)	16 (6.7%)
*Proteus* species	3 (0.9%)	3 (1.3%)
*Serratia marcescens*	14 (4.1%)	8 (3.4%)
*Streptococcus pyogenes*	1 (0.3%)	0

*L. pneumophila* was detected in five specimens, four with mixed detections, with *L. pneumophila* recovered in only one culture. No *C. pneumoniae* or *M. pneumoniae* were detected by the panel. Resistance genes were detected in 28 specimens. Sixteen were positive for *mecA/C* and MREJ with concordant growth of methicillin-resistant *S. aureus* (MRSA) in 13 cultures, 1 culture with methicillin-susceptible *S. aureus* growth, and 2 cultures with no *S. aureus* recovered. Ten specimens were positive for *bla*_CTX-M_ with concordant growth of cephalosporin-resistant *K. pneumoniae* or *E. coli* in eight cultures. One specimen was positive for *bla*_OXA-48-like_ and another for *bla*_KPC_ with no concordant growth in culture. *bla*_IMP_, *bla*_NDM_, and *bla*_VIM_ were not found in any specimen.

Viruses were detected by the panel in 41 specimens (1 with co-detection of rhino/enterovirus and RSV), including 26 rhinovirus/enterovirus, 5 parainfluenza, 3 RSV, 2 human metapneumovirus, 1 influenza A, 1 adenovirus, and 4 coronaviruses (non-SARS-CoV-2). In 20 specimens, only viruses were detected, with the remaining 21 having concomitant bacterial detections.

Culture positivity rate was 35.3% (199/563) overall, higher for pneumonia—compared to OP-NI-associated specimens (55.5% vs 18.8%). The most common bacteria detected were *S. aureus* (44.2%), *P. aeruginosa* (18.1%), and *E. coli* (10.6%). Detection of more than one bacterium was observed in 33.7% (67/199) specimens.

Concordance between BF-PP and culture is presented in Table 3. The lowest concordances were observed in the URM group (50% for *S. pneumoniae*, 25% for *H. influenzae*, and 17% each for *S. agalactiae* and *M. catarrhalis*). Cultures reported as usual respiratory microbiota in which URM group bacteria were detected by the BF-PP were re-reviewed by laboratory technologists in real time. *S. pneumoniae* was reported from culture and detected by the BF-PP in 10 specimens, with the panel alone being positive in additional 10 specimens. No cultures reported as usual respiratory microbiota from specimens testing positive for *S. pneumoniae* by the BF-PP were found to harbor *S. pneumoniae* after re-review of cultures. *H. influenzae* was detected in 15 of 60 specimens by the BF-PP and culture and by the panel alone in 31 of 60. In the remaining 14 of 60, *H. influenzae* was found after re-review of cultures in non-predominant quantities, not qualifying for specific reporting of *H. influenzae* according to the laboratory’s practices (i.e., reported as usual respiratory microbiota). *S. agalactiae* was detected by culture and the BF-PP in 5 of 30 specimens, with the panel alone being positive in an additional 17. In the remaining 8 of 30, *S. agalactiae* was found after re-review of cultures, in non-predominant quantities, thereby qualifying for reporting as usual respiratory microbiota. *M. catarrhalis* was detected by both tests in 2 of 12 specimens, with 5 being positive only by the panel. In the remaining 5, *M. catarrhalis* was found after re-review of cultures, in non-predominant quantities, thereby qualifying for reporting as usual respiratory microbiota (Table S2).

**TABLE 3:** Concordance rate between BIOFIRE FILMARRAY pneumonia panel and culture^*a*^

Microorganism	Concordance	PPA	95% CI	NPA	95% CI
*Staphylococcus aureus*	76/94 (81%)	89.5%	81.1%–95.1%	96.4%	94.4%–97.9%
*Streptococcus pneumoniae*	10/20 (50%)	100%	69.2%–100%	98.2%	96.7%–99.1%
*Haemophilus influenzae*	15/60 (25%)	93.8%	69.8%–99.8%	91.8%	89.2%–93.9%
*Streptococcus agalactiae*	5/30 (17%)	100%	47.8%–100%	95.5%	93.5%–97.1%
*Moraxella catarrhalis*	2/12 (17%)	100%	15.8%–100%	98.2%	96.8%–99.1%
*Pseudomonas aeruginosa*	33/38 (87%)	91.7%	77.5%–98.3%	99.1%	97.8%–99.7%
*Acinetobacter baumannii*	4/6 (67%)	100%	39.8%–100%	99.6%	98.7%–99.9%
*Escherichia coli*	18/21 (86%)	85.7%	63.7%–96.9%	99.5%	98.4%–99.9%
*Enterobacter cloacae*	11/13 (85%)	84.6%	54.6%–98.1%	99.6%	98.7%–99.9%
*Klebsiella aerogenes*	6/6 (100%)	85.7%	42.1%–99.6%	100%	99.3%–100%
*Klebsiella oxytoca*	5/6 (83%)	55.6%	21.2%–86.3%	99.8%	99.0%–100%
*Klebsiella pneumoniae*	15/20 (75%)	93.8%	69.8%–99.8%	99.1%	97.9%–99.7%
*Proteus* species	3/3 (100%)	100%	29.2%–100%	100%	99.3%–100%
*Serratia marcescens*	12/14 (86%)	80.0%	51.9%–95.7%	99.6%	98.7%–99.9%

^*a*^ Positive-percent agreement (PPA) and negative-percent agreement (NPA) for bacteria detected by the BIOFIRE FILMARRAY pneumonia panel. CI, confidence interval.

The overall PPA and NPA across all specimens was 91.4% (CI 86.3%–94.9%) and 83.6% (CI 79.5%–87.2%), respectively. For *S. aureus*, PPA was 89.5% (CI 81.1%–95.1%) and NPA 96.4% (CI 94.4%–97.9%). For the URM group, PPA was 96.7% (CI 82.8%–99.9%), and NPA was 86.8% (CI 83.7%–89.6%). For the non-fermenting Gram-negative bacilli group, PPA and NPA were 92.5% (CI 79.6%–98.4%) and 98.7% (CI 97.3%–99.5%), respectively. PPA for the Enterobacterales group was 84.6% (CI 54.6%–98.1%), and NPA was 99.6% (CI 98.7%–99.9%). The PPA and NPA for each microorganism are presented in Table 3.

For all the different microorganism groups, PPA was higher for pneumonia compared to OP-NI specimens (Table 4), with differences being most prominent for Enterobacterales (91.0% vs 50.0%) though not statistically significant (*P* = 0.29). For the URM group, PPA was numerically but not statistically higher in pneumonia compared to OP-NI [100% (CI 84.6%–100%) and 87.5% (CI 47.4%–99.7%), respectively, *P* = 0.09], whereas NPA was significantly higher in OP-NI [82.3% (76.8%–87.0%) vs 90.4% (CI 86.5%–93.5%), *P* = 0.006]. Table S3 provides PPA and NPA stratified by CAP, HAP, and VAP. Specimens from pneumonia stratified by BAL fluid vs other specimen types (non-BAL fluid), had higher PPA in BAL fluid except for the URM group (100% in both groups); for *S. aureus*, PPA was lower in BAL fluid [85.7% (CI 42.1%–99.6%) vs 90.6% (CI 79.3%–96.9%), *P* = 0.6]. NPA for the URM group was 79.9% (CI 73.4%–85.4%) for non-BAL fluid and 91.7% (CI 80.0%–97.7%) for BAL fluid specimens (*P* = 0.05). For all other groups of organisms, NPA was higher than 94% (Table 5).

**TABLE 4:** Positive-percent agreement (PPA) and negative-percent agreement (NPA) of BIOFIRE FILMARRAY pneumonia panel compared to culture for pneumonia and infection other than pneumonia or no infection specimens with 95% confidence interval (CI)

	Pneumonia				Infection other than pneumonia or no infection			
Microorganisms	PPA	95% CI	NPA	95% CI	PPA	95% CI	NPA	95% CI
*Staphylococcus aureus*	90.0%	79.5%–96.2%	95.4%	91.4%–97.9%	88.5%	69.9%–97.6%	97.2%	94.5-98.8%
Usual respiratory microbiota	100%	84.6%–100%	82.3%	76.8%–87.0%	87.5%	47.4%–99.7%	90.4%	86.5-93.5%
Non-fermenting Gram-negative bacilli	96.3%	81.0%–99.9%	97.8%	94.9%–99.3%	84.6%	54.6%–98.1%	99.3%	97.6-99.9%
Enterobacterales	91.0%	58.7%–99.8%	99.2%	97.1%–99.9%	50.0%	1.3%–98.7%	100%	98.8-100%

**TABLE 5:** Positive-percent agreement (PPA) and negative-percent agreement (NPA) of BIOFIRE FILMARRAY pneumonia panel compared to culture for bronchoalveolar lavage fluid and non-bronchoalveolar lavage fluid (sputum, induced sputum, tracheal secretions) specimens with 95% confidence interval (CI)

	Non-bronchoalveolar lavage fluid				Bronchoalveolar lavage fluid			
Microorganisms	PPA	95% CI	NPA	95% CI	PPA	95% CI	NPA	95% CI
*Staphylococcus aureus*	90.6%	79.3%–96.9%	94.7%	89.8%–97.7%	85.7%	42.1%–99.6%	97.7%	88.0%–99.9%
Usual respiratory microbiota	100%	82.4%–100%	79.9%	73.4%–85.4%	100%	29.2%–100%	91.7%	80.0%–97.7%
Non-fermenting Gram-negative bacilli	95.5%	77.2%–99.9%	97.2%	93.7%–99.1%	100%	47.8%–100%	100%	92.3%–100%
Enterobacterales	88.9%	51.8%–99.7%	99.0%	96.3%–99.9%	100%	15.8%–100%	100%	92.8%–100%

Table 6 summarizes BF-PP semiquantitative results (genomic abundance) for the different bacterial groups and culture findings. For *S. aureus*, 42.1% of specimens with low genomic abundance did not yield bacterial growth, and 47.4% showed low growth. Among specimens with high genomic abundance, 1.8% had no bacterial growth, while 71.4% demonstrated high growth. For the URM group, 25.5% of specimens with low genomic abundance did not grow any bacteria in cultures, while 68.6% were reported as usual respiratory microbiota; among specimens with high genomic abundance, 50.7% were classified as usual respiratory microbiota, with 36.6% showing high bacterial growth in cultures. For the non-fermenting Gram-negative bacilli group, 50.0% of low abundance specimens displayed low growth, and 35.7% were culture negative; in specimens with high genomic abundance, 6.7% were culture-negative, while 53.3% had high growth. For the Enterobacterales group, 27.3% of low abundance specimens had no growth, and 59.1% had low growth; among high abundance specimens, 61.5% had high growth, and only 2.6% were culture-negative.

**TABLE 6:** Correlation between genomic abundance detected by the BIOFIRE FILMARRAY pneumonia panel and growth in culture for each microorganisms group

Microorganism	Genomic abundance (Low [<10^6^]) (High [≥10^6^])	No growth in culture	Low growth in culture (+1, +2)	High growth in culture (+3, +4)	Reported as usual respiratory microbiota
*Staphylococcus aureus* (*N* = 94)	Low (*N* = 38)	16 (42.1%)	18 (47.4%)	4 (10.5%)	
	High (*N* = 56)	1 (1.8%)	15 (26.8%)	40 (71.4%)	
Usual respiratory microbiota group (*N* = 122)	Low (*N* = 51)	13 (25.5%)	2 (3.9%)	1 (1.9%)	35 (68.6%)
	High (*N* = 71)	7 (9.9%)	2 (2.8%)	26 (36.6%)	36 (50.7%)
Non-fermenting Gram-negative bacilli (*N* = 44)	Low (*N* = 14)	5 (35.7%)	7 (50.0%)	2 (14.3%)	
	High (*N* = 30)	2 (6.7%)	12 (40.0%)	16 (53.3%)	
Enterobacterales (*N* = 83)	Low (*N* = 44)	12 (27.3%)	26 (59.1%)	6 (13.6%)	
	High (*N* = 39)	1 (2.6%)	14 (35.9%)	24 (61.5%)	

Clinical consistency analysis between the BF-PP and culture for patients ultimately classified as having pneumonia showed 79.9% consistent, 5.9% partially consistent, and 14.2% inconsistent results. Those with CAP had the highest consistency rate of 83.0% and lowest inconsistency rate of 12.3%, compared to HAP (consistency 78.6%, inconsistency 14.3%) and VAP (consistency 76.0%, inconsistency 18.0%). Across all specimen types, most specimens were consistent. BAL fluid had the highest consistency of 90.2%, with a low partial consistency (3.9%) and inconsistency (5.9%) (Table 7). Specimens with genomic abundance of 10^6^ or greater had consistent and inconsistent rates of 81.1% and 7.5%, respectively, while specimens with low genomic abundance (<10^6^), were 69.2% consistent and 25.0% inconsistent. Specimens in which single microorganisms were detected were 82.5% consistent, and specimens with more than one microorganism detected were 70.1% consistent.

**TABLE 7:** Clinical consistency rate between the BIOFIRE FILMARRAY pneumonia panel and culture

		Consistent	Partially consistent	Inconsistent
Clinical syndrome	Pneumonia (*N* = 254)	203 (79.9%)	15 (5.9%)	36 (14.2%)
	Community-acquired pneumonia (*N* = 106)	88 (83.0%)	5 (4.7%)	13 (12.3%)
	Hospital-acquired pneumonia (*N* = 98)	77 (78.6%)	7 (7.1%)	14 (14.3%)
	Ventilator-associated pneumonia (*N* = 50)	38 (76.0%)	3 (6.0%)	9 (18.0%)
Specimen type	Sputum (*N* = 81)	64 (79.0%)	7 (8.7%)	10 (12.3%)
	Induced sputum (*N* = 14)	12 (85.7%)	0	2 (14.3%)
	Tracheal secretions (*N* = 108)	81 (75.0%)	6 (5.6%)	21 (19.4%)
	Bronchoalveolar lavage fluid (*N* = 51)	46 (90.2%)	2 (3.9%)	3 (5.9%)
Genomic copies	10^4^ (*N* = 27)	19 (70.4%)	1 (3.7%)	7 (25.9%)
	10^5^ (*N* = 25)	17 (68.0%)	2 (8.0%)	6 (24.0%)
	10^6^ (*N* = 26)	21 (80.8%)	3 (11.5%)	2 (7.7%)
	10^7^ (*N* = 80)	65 (81.3%)	9 (11.2%)	6 (7.5%)
	<10^6^ (*N* = 52)	36 (69.2%)	3 (5.8%)	13 (25.0%)
	≥10^6^ (*N* = 106)	86 (81.1%)	12 (11.3%)	8 (7.5%)
Number of microorganisms identified	None (*N* = 90)	76 (84.4%)	0	14 (15.6%)
	One (*N* = 97)	80 (82.5%)	2 (2.1%)	15 (15.4%)
	More than one (*N* = 67)	47 (70.1%)	13 (19.4%)	7 (10.4%)

## DISCUSSION

BF-PP is a syndromic multiplex PCR assay designed to detect a broad range of microorganisms with a short turn-around time. Here, performance was compared to conventional cultures in patients with suspected pneumonia. Several studies have been conducted on patients with specific syndromic respiratory infections and specimen types, primarily focusing on tracheal secretions and BAL fluid (6, 12–16). In contrast, this study included three specimen types obtained from a heterogeneous group of patients, including those on whom cultures were ordered, but who were ultimately determined not to have pneumonia, reflecting a real-world scenario.

Overall positivity rate of the BF-PP was 44.8%. Although this is higher than reported for conventional methods (4, 5), it is relatively low. This can be partially explained because nearly half of specimens were from patients who ultimately were not considered to have pneumonia. Focusing on specimens from those ultimately considered to have pneumonia supports this explanation, as the positivity rate in this subgroup approached 65%, higher than the positivity rate among those without pneumonia. The positivity rate for pneumonia-associated specimens was higher for sputum (63%) compared to BAL fluid (45%), consistent with previous reports (6, 8).

The most frequent bacterium detected in specimens from patients ultimately considered to have pneumonia was *S. aureus* (27%). This aligns with studies from Europe and the United States, where *S. aureus* was among the most detected microorganisms, with similar detection rates (20%–28%) (17, 18). Whether this reflects *S. aureus* being a pathogen or a colonizer is difficult to ascertain; if the former is the case, *S. aureus* would be the most common cause of pneumonia. It may be hypothesized that there has been an increase in *S. aureu*s pneumonia, given that the study period was during the COVID-19 pandemic and that *S. aureus* may play a role in post-viral pneumonia complications (19). *S. pneumoniae*, once considered the most frequent bacterial cause of pneumonia, was detected in only 18 (7.6%) pneumonia-associated specimens, consistent with recent studies reporting a decline in pneumococcal pneumonia, such that it comprises only 5%–9% of CAP (1, 5).

The introduction of sensitive molecular diagnostics provides insight into the potential role of viruses in pneumonia. A meta-analysis on CAP demonstrated viral detection in 9%–56% of respiratory specimens (20). Another study reported a detection rate of 22% in HAP (21). In the current study, viral detection was relatively low at 7.5%, likely impacted by the COVID-19 pandemic, which may have affected the prevalence of other viruses.

Discrepancy between the BF-PP and cultures was greatest for the URM group of bacteria. Initially, it was speculated that this could be due to the laboratory’s reporting method, which categorized usual respiratory microbiota without identifying microorganisms when no predominant growth was observed. However, upon further examination considering any identification of these specific species in cultures, the discrepancy remained. This may be attributed to the fastidious nature of these organisms or to prior antibiotic exposure, which could have inhibited bacterial growth, resulting in fewer positive cultures (12, 17). That said, the clinical significance of BF-PP positive/culture negative URM group bacterial detection is sometimes unclear.

Overall PPA and NPA were comparable to previous studies (6, 18, 22). For Gram-negative bacteria, the PPA for the Enterobacterales group was lower (85%) compared to the non-fermenting Gram-negative bacilli group (93%). This difference could be mistakenly interpreted as poorer performance of the BF-PP in detecting Enterobacterales, with a higher number of false negatives for this group (i.e., a greater proportion of isolates recovered by cultures but not detected by the BF-PP). However, this may be a result of the low prevalence of these bacteria in the specimen types evaluated, rather than a high rate of actual false negatives.

The PPA was higher in specimens from pneumonia patients for each group of microorganisms, particularly for the Enterobacterales group, although this difference was not statistically significant. The NPA for the URM group in pneumonia and non-pneumonia specimens was lower compared to other groups of bacteria, with a significantly lower NPA in the pneumonia group. This could be related to the high discrepancy between the panel and cultures for these specific species, as discussed earlier. Overall, performance was better in BAL fluid compared to all other specimen types pooled together, particularly in terms of the NPA for the URM group. This suggests a reduction in “background noise” from potentially colonizing microorganisms and a higher predictive value for pneumonia pathogens in BAL specimens compared to other specimen types.

Previous studies have assessed the correlation between BF-PP semiquantitative bacterial abundance and bacterial growth in culture; some have demonstrated a strong relationship (7, 12, 23), emphasizing the importance of reporting these data for clinical interpretation (16). In this study, distinct patterns were revealed across different bacterial groups. Specimens with higher genomic abundance were more likely to exhibit higher growth in culture, while those with lower genomic abundance showed lower growth or negative cultures. For *S. aureus*, there was a strong correlation between high genomic abundance and culture growth, with 71% of high-abundance specimens exhibiting high growth and only 2% being culture-negative. Similar trends were observed in the Enterobacterales and non-fermenting Gram-negative bacilli groups although correlation was weaker for the latter. However, this relationship was not observed in the URM group, in which most specimens with high genomic abundance were classified as usual respiratory microbiota and only 37% showed high growth in culture. This suggests that while high genomic abundance is generally associated with increased likelihood of bacterial growth, which could indicate active infection or significant colonization, this pattern does not apply to bacteria that are part of the normal respiratory microbiota. This highlights an interpretive challenge when URM group bacteria are detected by the BF-PP.

While the definition of diagnostic accuracy of the BF-PP is expanding, its clinical utility is beginning to be addressed (12, 22, 24). In our recently published RCT (9), findings indicated that BF-PP use was associated with a shorter time to antibiotic escalation, faster de-escalation of Gram-positive antibiotics, and decreased intensive care unit admissions among pneumonia patients. In the current study, the clinical consistency rate was calculated by answering the question as to whether, for a patient with pneumonia, comparing BF-PP results with culture would lead to the same clinical decision, particularly regarding antibiotic modification. The overall consistency was 80%, highest in CAP, in BAL specimens and in cases with high genomic abundance. Reasons for inconsistency or partial consistency included the growth of bacteria not included in the panel, such as *Stenotrophomonas maltophila*, or the detection of an organism that would impact antibiotic treatment (such as MRSA) by one test and not the other.

This study has limitations. First, most specimens were obtained from patients who had been exposed to antibiotics prior to specimen collection. This could have affected the yield of cultures, rendering them less optimal as a comparator and making it difficult to assess potential false positivity of the BF-PP. Second, there was no additional assay for comparison to resolve discrepant results between the panel and cultures. Although AST enabled assessment of genetic resistance determinants, the low number of resistant bacteria detected, particularly Gram-negative bacteria, may underestimate the clinical significance of the BF-PP as a diagnostic tool for guiding antimicrobial stewardship. This limitation affects the external validity of the findings in regions with different epidemiology, such as countries with higher rates of antimicrobial resistance, where this tool could potentially reduce the use of last-resort antibiotics.

Has the BF-PP changed the understanding of the etiology of pneumonia? Undoubtedly, the introduction of molecular methods allows more rapid diagnosis of a broad range of microorganisms potentially implicated in pneumonia (1). However, distinguishing between true pathogens, usual respiratory microbiota, and colonizing organisms remains a challenge, representing the Achilles’ heel of pneumonia diagnosis. The challenge extends beyond the false positivity of the assay to include a high rate of negative results in pneumonia cases. A study using multiple microbiologic methods in an intense search for an etiologic agent failed to detect a pathogen in 55% of CAP cases (25). In the current study, 35% of pneumonia specimens did not yield any microorganism. Reasons for this remain unclear, but potential explanations include microorganisms not detected by the BF-PP, including the possibility that some cases are caused by microorganisms that have yet to be discovered or considered pathogenic in the context of pneumonia, the impact of prior antibiotic exposure, or the possibility that some cases are actually non-infectious conditions mimicking pneumonia.

In conclusion, the BF-PP has reliable analytical performance and provides a high rate and rapid detection of microorganisms in respiratory specimens, offering advantages over cultures. It could serve as an adjunct to guide early clinical decisions, facilitating antimicrobial stewardship. However, challenges in distinguishing between true pathogens and colonizing organisms may confound clinical interpretation, requiring careful consideration in clinical decision-making. Finally, despite the use of this advanced diagnostic tool, one-third of pneumonia cases remained without a microbiologic etiology.

## Data Availability

Data for this study can be made available upon request to the corresponding author after publication, subject to review of the request details, approval from the institutional research board, and the completion of a signed data access agreement.

## SUPPLEMENTAL MATERIAL

Supplemental tables spectrum.00571-25-s0001.pdf Tables S1 to S4.

ASM does not own the copyrights to Supplemental Material that may be linked to, or accessed through, an article. The authors have granted ASM a non-exclusive, world-wide license to publish the Supplemental Material files. Please contact the corresponding author directly for reuse.
