# Supplementary material for: Performance of BIOFIRE FILMARRAY pneumonia panel in suspected pneumonia: insights from a real-world study
Source: Microbiol Spectr. 2025 May 22;13(7):e00571-25. doi: 10.1128/spectrum.00571-25 (PMC12210945; doi:10.1128/spectrum.00571-25)
Supplement: Supplemental tables — Tables S1 to S4. [file spectrum.00571-25-s0001.pdf]

Table S1. BIOFIRE® FILMARRAY® Pneumonia Panel *versus* culture and consistency classification.

**Number of microorganisms detected**	**BIOFIRE® FILMARRAY® Pneumonia Panel**	**Culture**	**Consistency**
None	Negative	Negative	Consistent
		Usual respiratory microbiota	Consistent
		Staphylococcus aureus	Inconsistent
		Gram negative bacteria (Enterobacterales or non-fermenting Gram-negative bacilli, including those not included in the panel)	Inconsistent
One	Gram negative bacteria (Enterobacterales/non-	The same organism with or without usual respiratory microbiota	Consistent

	fermenting Gram-negative bacilli) or *S. aureus*	The same organism with different organism that is not usual respiratory microbiota	Partially consistent
		Negative or usual respiratory microbiota	Inconsistent
	Virus	Negative or usual respiratory microbiota	Consistent
		Bacteria other than usual respiratory microbiota	Inconsistent
	Member of usual respiratory microbiota	Same organism or usual respiratory microbiota	Consistent
		Negative	Inconsistent
Two or more	Gram negative bacteria (Enterobacterales/non-	The same organisms with or without usual respiratory microbiota	Consistent

	fermenting Gram-negative bacilli) or *S. aureus*	Only one recovered or recovered with other bacteria not included in the panel	Partially consistent
	Gram negative bacteria (Enterobacterales/non-fermenting Gram-negative bacilli) or *S. aureus* with microorganism that is part of usual respiratory microbiota	Same Gram-negative bacterium (Enterobacterles/non-fermenting Gram-negative bacilli) or *S. aureus* with or without usual respiratory microbiota	Consistent
	Usual respiratory microbiota	Usual respiratory microbiota or negative	Consistent

Table S2. Correlations between the BIOFIRE® FILMARRAY® Pneumonia Panel and culture for the 'Usual respiratory microbiota' group.

Microorganism	Discrepancy rate	Panel +/culture+	Panel +/culture -	Panel +/culture reported 'usual respiratory microbiota' and positive after re-review
Streptococcus pneumoniae (N=20)	50%	10	10	0
Haemophilus influenzae (N=60)	75%	15	31	14
Streptococcus agalactiae (N=30)	83%	5	17	8
Moraxella catarrhalis (N=12)	83%	2	5	5

N= number of positive specimens by the BIOFIRE® FILMARRAY® Pneumonia Panel for each microorganism.

Table S3. Positive-percent agreement (PPA) and negative-percent agreement (NPA) for community-acquired, hospital-acquired, and ventilator-associated pneumonia specimens with 95% confidence intervals (CIs).

	**Community-acquired pneumonia**				**Hospital-acquired pneumonia**				**Ventilator-associated pneumonia**			
**Microorganism**	**PPA**	**95% CI**	**NPA**	**95% CI**	**PPA**	**95% CI**	**NPA**	**95% CI**	**PPA**	**95% CI**	**NPA**	**95% CI**
Staphylococcus aureus	85.7%	63.7-96.9%	98.8%	93.6-99.9%	91.3%	72.0-98.9%	93.3%	85.1-97.8%	93.8%	69.8-99.8%	91.2%	76.3-98.1%
Usual respiratory microbiota	100%	69.2-100%	77.1%	67.4-85.1%	100%	47.8-100%	86.0%	77.3-92.3%	100%	59.0-100%	86.1%	72.1-94.7%
Non-fermenting Gram-negative bacilli	91.7%	61.5-99.8%	98.9%	94.2-99.9%	100%	71.5-100%	96.6%	90.3-99.3%	100%	39.8-100%	97.8%	88.5-99.9%
Enterobacterales	100%	15.8-100%	99.0%	94.8-99.9%	85.7%	42.1-99.6%	98.9%	94.0-99.9%	100%	15.8-100%	100%	92.6-100%

Table S4. Distribution of genomic abundance for the 'usual respiratory microbiota' group in discrepant specimens (positive BIOFIRE® FILMARRAY® Pneumonia Panel / negative culture).

Microorganism	**10^4**	**10^5**	**10^6**	**$\geq 10^7$**
Streptococcus pneumoniae	4 (40%)	2 (20%)	1 (10%)	3 (30%)
Streptococcus agalactiae	9 (36%)	7 (28%)	4 (16%)	5 (20%)
Haemophilus influenzae	11 (24.4%)	9 (20%)	6 (13.3%)	19 (42.2%)
Moraxella catarrhalis	0	6 (60%)	0	4 (40%)
